# Evaluation of Parotid Salivary Glucose Level for Clinical Diagnosis and Monitoring Type 2 Diabetes Mellitus Patients

**DOI:** 10.1155/2017/2569707

**Published:** 2017-01-31

**Authors:** Beibei Wang, Juan Du, Zhao Zhu, Zhihong Ma, Songlin Wang, Zhaochen Shan

**Affiliations:** ^1^Oral and Maxillofacial Surgery Department, School of Stomatology, Capital Medical University, Beijing, China; ^2^Beijing Coloproctological Hospital (Beijing Erlonglu Hospital), Beijing, China

## Abstract

*Background*. To investigate the relationships among blood glucose, mixed saliva glucose, and parotid glucose in type 2 diabetes patients and to evaluate the diagnostic and monitoring value of salivary gland glucose in patients with type 2 diabetes (type 2DM).* Material and Methods*. Thirty patients with type 2DM and 30 healthy age- and sex-matched individuals were included in this study. Glucose levels in unstimulated mixed saliva and in unstimulated parotid saliva were measured by the glucose oxidase peroxidase method.* Results*. The blood glucose and parotid salivary glucose levels in type 2DM patients were significantly higher than those in the controls (*P* < 0.05). The blood glucose, parotid salivary glucose, and mixed salivary glucose were 7.46 ± 1.44 mmol/L, 0.18 ± 0.19 mmol/L, and 3.17 × 10^−2^ ± 2.84 × 10^−2^ mmol/L, respectively, in the type 2DM group; the corresponding glucose levels in the control group were 5.56 ± 0.71 mmol/L, 7.70 × 10^−2^ ± 6.02 × 10^−2^ mmol/L, and 3.47 × 10^−2^ ± 2.79 × 10^−2^ mmol/L. The parotid salivary and blood glucose levels in type 2DM patients were strongly correlated; the linear regression equation for blood glucose and parotid salivary glucose was *Y* = 6.267*X* + 6.360, with *r* = 0.810. However, mixed salivary glucose levels were not significantly different in the type 2 diabetes group compared with the control group.* Conclusion*. Our results suggest that parotid salivary glucose has potential as a biomarker to monitor type 2DM and as a painless, noninvasive method for the management of type 2DM.

## 1. Introduction

Diabetes mellitus (DM) is a metabolic syndrome characterized by hyperglycemia and disturbances in the metabolism of carbohydrates, proteins, and lipids [1]. It is an endocrine disease characterized by a deficit in the production of insulin, which is responsible for lowering blood glucose concentration. Two etiologies of DM are recognized, type 1 and type 2 [2, 3]. Type 1 diabetes is characterized by an absolute deficiency of insulin owing to the destruction of pancreatic beta cells, while type 2 is caused primarily by insulin resistance in peripheral target organs, such as the liver, muscle, and adipose tissue [1, 4].

The global prevalence of diabetes in the adult population is currently estimated at 6.4%, and the number of people with diabetes is estimated to rise from 180 million in the year 2000 to 320 million in 2025 [5]. China, with 90 million people suffering from diabetes in 2010, has the largest diabetes population in the world, comprising 9.7% of the nation's adult population. Currently, diabetes is diagnosed by measuring blood glucose levels, and, because this generally requires a finger stick or venipuncture, it is physically and psychologically traumatic to the patients. Therefore, a simple, noninvasive, and painless procedure, such as measuring salivary glucose concentration, would be desirable.

Saliva is a biological fluid that can reflect local and systemic changes because the composition of saliva is influenced by the hormonal, immunologic, neurologic, nutritional, and metabolic state of the individual [6, 7]. The incidence of caries, periodontal disease, and candidosis may be increased in individuals with elevated blood glucose levels [8, 9]. Higher salivary glucose levels have been reported in diabetes patients compared with the levels in nondiabetics [9–12]. However, reports of the comparative values of blood and salivary glucose are inconsistent [13, 14]. These discrepancies may result from different population samples and use of different methods for collecting saliva or for glucose analysis. The poor correlation between blood and saliva glucose concentrations often seen in diabetes patients could result from oral retention of alimentary carbohydrates, glucose utilization by oral bacteria, release of carbohydrates from salivary glycoproteins, and contamination of saliva by a large outflow of crevicular fluid in patients with a poor gingival status [15]. However, saliva can be directly collected from the parotid glands using a Lashley cup [16] without being released into the oral cavity [17].

A previous study found that the glucose concentration in parotid secretions correlated differently with plasma glucose concentrations for each subject and at a much lower level compared with blood glucose. Although the mean glucose concentration in the parotid saliva of subjects with diabetes was slightly higher at each measurement compared with that of subjects without diabetes, there was considerable overlap of the concentration curves for individuals in each group. Therefore, further study of the correlation between parotid and plasma glucose concentrations is important because of the potential value in the diagnosis and monitoring of diabetes mellitus [18]. The present study was a comparative analysis of the glucose concentrations in mixed saliva from all salivary glands (i.e., saliva secreted into the oral cavity), unstimulated parotid glands, and blood glucose in patients with type 2DM and subjects without diabetes.

## 2. Materials and Methods

### 2.1. Subjects

The study was approved by Beijing Stomatologic Hospital ethics committee (number: 2014024), and all participants gave their informed consent. Thirty patients (13 male and 17 female) with type 2DM from 49 to 82 years of age (mean, 68.3 ± 9.4 years) and a control group of 30 clinically healthy subjects (15 male and 15 female) from 47 to 84 years of age (mean, 67.5 ± 9.1 years) were enrolled. All the participants were recruited at the Department of Geriatric Dentistry, Beijing Stomatologic Hospital, Capital Medical University between 2014 and 2016.

Individuals who smoked, suffered from alcoholism, were pregnant, were edentulous, had prior surgery of the salivary glands, and were being treated with radiotherapy of the head and neck region and those with Sjögren's syndrome, rheumatoid arthritis, or lupus erythematosus were excluded from this study. The control subjects were free of chronic diseases and did not take any medications other than vitamins or occasional analgesics.

### 2.2. Sample Collection

Resting saliva from the parotid gland and venous blood (5 ml) were collected between 8:00 and 11:00 in the morning after an 8-hour fast; patients did not perform any oral hygiene in the 90 min before the saliva collection. The parotid duct orifice was visualized with a mouth mirror and rinsed with water. The parotid region was gently massaged until a small amount of clear fluid flew from the parotid gland duct. Then parotid saliva was collected using a Lashley cup in 10 min [16].

### 2.3. Mixed Saliva Collection

Subjects were asked to spit out or swallow their saliva and then stay still, passively allowing the saliva to drain over the lower lip into a preweighed test tube for 10 minutes.

### 2.4. Salivary Glucose Assay

The collected parotid saliva and mixed saliva were stored at −80°C until use. The glucose oxidase peroxidase method (GOD-POD) was used to determine the salivary glucose level. Briefly, saliva samples were thawed and centrifuged at 3000 rpm for 5 minutes, 4 *μ*L of the supernatant was transferred into Eppendorf tubes, and 2000 *μ*L of enzyme reagent from the glucose test kit (Leadman Biochemistry Co., Ltd, Beijing, China) was added to each sample. Three standards were also prepared. The salivary glucose assay mixtures were incubated in a water bath at 37°C for 5 min and were then transferred to 1.5 mL cuvettes. The absorbance was read at a wavelength of 505 nm with a spectrophotometer and the glucose level was determined by comparison to the standard solution.

Fasting blood glucose was tested before breakfast using a glucose meter (Roche Ltd, Switzerland). Briefly, the index finger was disinfected with 70% alcohol, and a disposable sterile needle was used to obtain a drop of blood, which was collected on a glucose test strip and then inserted into the glucose meter. The blood glucose level was determined and recorded on the patient's chart.

### 2.5. Statistics Analysis

All statistical calculations were performed using SPSS v.12 statistical software. Student's *t*-test was used to determine statistical significance, which was indicated by *P* < 0.05 or *P* < 0.01.

## 3. Results

### 3.1. Salivary Flow in DM Group and Control Group

Mixed salivary flow was 0.32 ± 0.15 ml/min and 0.29 ± 012 ml/min in patients with type 2 diabetes and the healthy subjects, respectively; the flow of mixed saliva was not significantly different in two groups. The level of the parotid salivary flow was not significantly different in patients (0.05 ± 0.03 ml/min) with type 2 diabetes and control group (0.04 ± 0.03 ml/min).

### 3.2. Glucose Levels in Blood and Saliva

To evaluate the diagnostic and monitoring value of single salivary gland glucose in patients with type 2 diabetes, we first determined the levels of blood, mixed salivary, and parotid salivary glucose in type 2 diabetes patients. The mean levels of blood, parotid salivary, and mixed salivary glucose were 7.46 ± 1.44 mmol/L, 0.18 ± 0.19 mmol/L, and 3.17 × 10^−2^ ± 2.84 × 10^−2^ mmol/L in the type 2DM group and 5.56 ± 0.71 mmol/L, 7.70 × 10^−2^ ± 6.02 × 10^−2^ mmol/L, and 3.47 × 10^−2^ ± 2.79 × 10^−2^ mmol/L in the healthy subjects, respectively (Table 1). The blood glucose and parotid salivary glucose levels were significantly higher in the type 2DM group than in the control group. However, the mixed salivary glucose levels in the type 2DM and control groups were not significantly different (Table 1). Interestingly, the parotid salivary glucose level was significantly higher than the mixed salivary glucose level in both the type 2DM and control groups (Table 1). These results support the potential value of parotid saliva as a biomarker for monitoring diabetes mellitus.

### 3.3. The Correlation of Blood Glucose and Parotid Salivary Glucose

**Figure 1:**
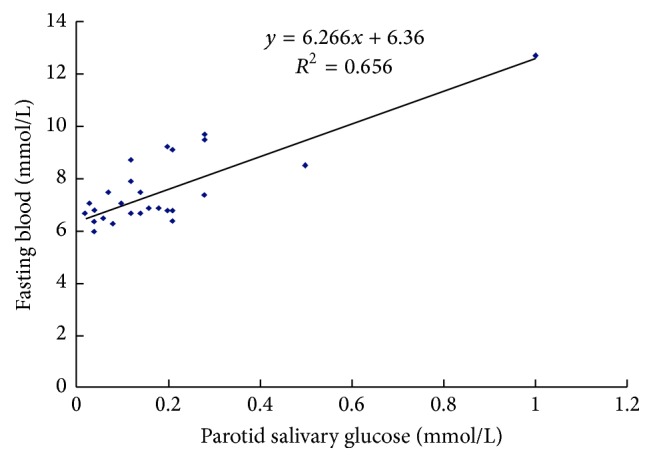
The correlation between parotid salivary glucose and blood glucose in DM group, correlation coefficient *r* = 0.810.

To assess the correlation of parotid salivary glucose with blood glucose levels in people with diabetes, we performed a regression analysis. As shown in Figure 1, the linear regression equation for parotid salivary glucose and blood glucose was *Y* = 6.267*X* + 6.360, *r* = 0.810, *p* = 0.000. The results of linear regression indicated a significant relationship between the levels of parotid salivary glucose and blood glucose in the diabetes patients.

## 4. Discussion

Saliva, like plasma or serum, is a unique, complex body fluid, and the adequate production of saliva is essential for maintaining oral health. Normally, about 1000–1500 ml of saliva is produced daily. Saliva is more than 99% water and contains sodium, potassium, glycoproteins, glucose, amino acids, and a variety of other substances. Previous studies have described the usefulness of saliva for the diagnosis of oral or systemic diseases such as periodontal diseases, oral squamous cell carcinoma, and human immunodeficiency virus (HIV) infection [19–21]. Advances in diagnostic technologies hold tremendous promise for achieving the long-term goal of developing clinically validated, saliva-based tests for health surveillance and early detection of oral disease and other systemic conditions.

In the present study, we found that the glucose levels in parotid saliva glucose were higher than in mixed saliva in both diabetes patients and controls. The blood glucose and parotid salivary glucose levels in type 2 diabetes patients were significantly higher than levels in the control group, and the glucose levels in parotid saliva were strongly correlated with the blood glucose levels in type 2 diabetes patients. However, there were no significant differences in the glucose levels in mixed saliva from diabetes patients and healthy controls after an 8-hour fast. These findings differ to some extent from those of other studies [22, 23]. A previous study by Darwazeh et al. showed that, in diabetes patients, salivary glucose concentration was significantly higher than in controls and was directly related to blood glucose concentration [22]. These discrepancies may be related to the condition of the subjects included in the study. In our study, we included type 2DM patients with routine medication control, which may have resulted in the low blood and salivary glucose concentrations observed. In another study, no correlation was found between saliva and plasma glucose levels [10]. Nonetheless, our study revealed a significant, strong correlation between parotid salivary and blood glucose levels but not between mixed salivary and blood glucose levels; the glucose level in parotid saliva but not that in mixed saliva may thus reflect the blood glucose level.

The advantages of salivary assessment include noninvasive collection and cost effectiveness for screening of large populations [17]. Saliva is currently recognized as an excellent diagnostic biomarker of human body characteristics. The saliva microbiome, for example, is reputed to be biometrically as accurate as a fingerprint when used for screening characteristics for normality [21]. There is a possibility that saliva could be used as an alternative for blood in some laboratory tests, for example, to detect viral infections, such as HIV and HCV, as a noninvasive procedure and allowing multiple sampling [24, 25].

Either single or mixed saliva can be collected, and it should be noted that many unknown factors and unstable elements influence the properties of mixed saliva. Saliva collected directly from a single gland is stable and not influenced by oral conditions; thus it might accurately reflect the status of blood glucose. Parotid saliva is easy to collect by both unstimulated and stimulated conditions (by chewing or acids). Dhanya and Hegde [9] reported that the salivary glucose concentration was higher when saliva was collected under unstimulated than under stimulated conditions. Other studies found no significant differences in glucose concentrations in saliva collected under unstimulated and stimulated conditions [13, 14]. Since patients may not readily accept chewing or acid stimulation, and the moisture content is higher in stimulated saliva; unstimulated saliva may be more representative of the normal physiological state. In this study, we collected parotid and mixed saliva from type 2DM patients and healthy subjects under unstimulated conditions.

In our study, we found the salivary glucose level was higher in the type 2DM group than in the control group. These results are in line with studies of the ultrastructure of rat salivary glands in an experimental diabetes model. The parotid gland cells had swollen intracytoplasmic mitochondria without visible cristae. Mitochondria are the primary structures of aerobic oxidation; thus, intracellular glucose aerobic oxidation was disrupted in this diabetes model [26]. Another study showed that the permeability of the parotid basal cell membrane was increased in diabetic patients, resulting in substantial glucose oversecretion from the parotid duct [27]. That may be the reason for the increased glucose concentration in parotid saliva of type 2DM patients. However, there are still many issues that need to be addressed and further explored. For example, how to improve the sensitivity for glucose detection due to its low level in saliva as compared to serum and to standardize sample collection methods prior to analysis. Therefore, it is important to develop more sensitive methods for detecting salivary glucose that are suitable for convenient self-management by DM patients.

## 5. Conclusion

Our results demonstrated that parotid salivary glucose has potential as an indicator to monitor type 2DM and a noninvasive method in the management of diabetic patients.

## Figures and Tables

**Table 1:** The blood glucose, parotid salivary, and mixed salivary glucose results in DM and control groups.

	DM group (*n* = 30)	Control group (*n* = 30)	*p*
Blood glucose	7.46 ± 1.44	5.56 ± 0.71	0.000
Parotid salivary glucose	0.18 ± 0.19	7.70 ± 6.02 × 10^−2^	0.01
Mixed salivary glucose	3.17 ± 2.84 × 10^−2^	3.47 ± 2.79 × 10^−2^	0.681

The units of glucose: mmol/L.
